# Validity and reliability of the Arabic community integration questionnaire in a Lebanese sample of adults with physical disability

**DOI:** 10.1371/journal.pone.0336717

**Published:** 2025-11-18

**Authors:** Nour El Hoda Saleh, Ali Orabi, Sleiman Fneish, Bilal Cheaito, Ibrahim Naim

**Affiliations:** 1 Department of Research, Health, Rehabilitation, Integration, and Research Center (HRIR), Beirut, Lebanon; 2 Doctoral School of Sciences and Technology, Lebanese University, Hadat, Lebanon; 3 Physical Therapy Department, Health, Rehabilitation, Integration, and Research Center (HRIR), Beirut, Lebanon; 4 Physical Medicine and Rehabilitation Department, Health, Rehabilitation, Integration, and Research Center (HRIR), Beirut, Lebanon; 5 Rehabilitation Nursing Department, Health, Rehabilitation, Integration, and Research Center (HRIR), Beirut, Lebanon; Novum Research and Innovation Group, LEBANON

## Abstract

**Background:**

Community integration is a critical factor influencing the quality of life for individuals with physical disabilities. However, there is a lack of culturally adapted tools to assess community integration in Arabic-speaking populations. This study aimed to translate, validate, and examine the psychometric properties of the Arabic version of the Community Integration Questionnaire (AR-CIQ) among Lebanese adults with physical disabilities.

**Methods:**

A total of 150 individuals with various physical disabilities participated in the study. The AR-CIQ was translated and adapted to the Lebanese cultural context. Exploratory Factor Analysis (EFA) was conducted to determine the factor structure, while reliability was assessed using Cronbach’s alpha coefficients. Correlations between community integration and quality of life (QOL) were also explored using the SF-12 questionnaire.

**Results:**

The EFA revealed a four-factor structure of the AR-CIQ, which explained 68.27% of the total variance. These factors included social outdoor integration, productive and social management, domestic integration, and social support. The reliability of the AR-CIQ subscales varied, with the domestic integration subscale showing excellent internal consistency (α = 0.830). Correlation analysis revealed significant associations between community integration and QOL, with the mental health component (MCS-12) showing a moderate positive correlation with community integration (r = 0.418, p < 0.01).

**Conclusion:**

The AR-CIQ is a valid and reliable tool for assessing community integration in Lebanese adults with physical disabilities. Its factor structure reflects the unique cultural and contextual aspects of Lebanon. This study highlights the need for culturally sensitive tools in disability research and suggests that enhancing community integration could improve mental health outcomes for individuals with physical disabilities. Future research should explore longitudinal outcomes and the effectiveness of community-based interventions to further support the integration of individuals with disabilities in Lebanese society.

## Introduction

Community Integration is considered the main goal of rehabilitating people with physical disabilities [1]. Following an acute injury or disease, individuals often experience impairments and activity limitations that lead to participation restrictions in various aspects of life, including social settings, domestic life, leisure activities, and educational or work pursuits [2]. The International Classification of Functioning, Disability and Health (ICF) provides a framework for understanding these concepts: activity limitations refer to an individual’s difficulties in performing tasks, while participation restrictions are problems a person may encounter in life situations, such as community involvement [3].

Research has identified numerous factors associated with community participation, including secondary complications of the injury or disease, such as mobility and cognitive function, as well as demographic characteristics like age and cultural background. Socioeconomic factors, including educational attainment, economic status, and the availability of social support, also play a significant role [4,5].

Over recent decades, the definition of community integration has been a major focus in rehabilitation research [6,7]. The concept is multidimensional, encompassing an individual’s ability to function and perform life roles across three key areas: independent living, social and leisure activities, and productive activities like work. Several tools have been developed to measure community integration, including the Reintegration to Normal Living Index [8], the Community Integration Measure [9], the Craig Handicap Assessment and Reporting Technique [10], and the Community Integration Questionnaire (CIQ) [11].

The CIQ was originally established for use in patients with traumatic brain injury (TBI) [11], and is now considered one of the most commonly known and widely used outcome measures of community integration and participation in people with different types of disabilities [12–14]. While other tools exist (e.g., the Reintegration to Normal Living Index, the Community Integration Measure, and the Craig Handicap Assessment and Reporting Technique), the CIQ was selected because it is one of the most established measures and evaluates involvement across three critical, distinct domains: home integration, social integration, and productivity [15]. Crucially, the CIQ is consistent with the World Health Organization’s concept of handicap, as it specifically evaluates integration at the participation level [14]. Its psychometric properties have been assessed across various populations and languages, including Spanish, Italian, Persian, and Chinese [16–19]. However, community integration and participation are not universal concepts; they are profoundly shaped by cultural and social contexts [19]. Currently, there is no validated Arabic version of the CIQ, and its psychometric properties have never been evaluated in a Lebanese population. The purpose of this study is to address this gap by translating and cross-culturally adapting the CIQ from English to Arabic. The study will also evaluate the psychometric properties of the adapted version, including its reliability, construct, and convergent validity, specifically within a Lebanese sample of adults with physical disabilities.

## Materials and methods

### Translation process

The translation process adhered to the five-step Guidelines for the process of cross-cultural adaptation of self-report measures [20]. Initially, two independent Lebanese native speakers accomplished the forward translation of the CIQ from English into the Arabic language. The first was a psychologist with clinical experience in the field of rehabilitation, and the second was a sworn translator without any medical background. Translators were asked to emphasize clarity and cultural appropriateness for the Lebanese context. Then a unified Arabic version of the CIQ was proposed by the two translators through consensus. The back translation procedure was conducted by two blinded, independent English native speakers. Both back-translated versions were compared to the original English version of the CIQ to ensure that the scale items were appropriately translated. Finally, a panel of experts consisting of translators, a physical medicine and rehabilitation doctor, a rehabilitation nurse, and a clinical psychologist met to consolidate the translated versions and agreed on a pre-final version of the Arabic CIQ (AR-CIQ).

### Pilot testing

A pilot study was conducted to assess the clarity and comprehensibility of the pre-final version of the AR-CIQ. A random sample of 20 Lebanese participants with physical disabilities was selected for this purpose. The participants had a mean age of 43.19 ± 10.55 years, and 12 of them were male. The sample included a diversity of diagnoses, such as: 7 participants having traumatic brain injury, 6 participants with spinal cord injury, and 5 participants with traumatic unilateral lower limb amputation. Participants were asked to provide feedback on any items they found unclear. Following the pilot study, they reported that the content was comprehensible and no adjustments were needed. Therefore, no modifications were made to the questionnaire based on the findings of the pilot study, indicating that the translated CIQ was considered clear and understandable by the participants.

### Study design and participants

A cross-sectional study was conducted among Lebanese adults with physical disabilities from various governorates throughout Lebanon between March 03, 2024, and June 20, 2024. Participants were selected based on the following inclusion criteria: diagnosed with a physical disability from the following causes: “Stroke, TBI, Spinal cord injury (SCI), Multiple sclerosis (MS), amputation, Spina bifida, or cerebral palsy”, aged 18 or older, and able to provide informed consent. Exclusion criteria included cognitive impairments that could hinder questionnaire comprehension. Participation was completely voluntary.

### Sample size calculation

The minimum required sample size for validating the 15-item Community Integration Questionnaire (CIQ) was determined to be 75 participants, based on the recommended ratio of 5 subjects per scale item for instrument validation studies [21]. However, to enhance the statistical power and generalizability of our findings, we intentionally aimed for a larger sample size. Our final sample included 150 participants, which corresponds to a more robust 10:1 ratio of subjects to scale items. This larger sample size is crucial for ensuring the stability of psychometric analyses, particularly for evaluating the instrument’s factor structure and convergent validity, and for providing more reliable results that can be broadly generalized to the population of adults with physical disabilities in Lebanon.

### Ethical considerations

The study protocol was approved by the Institutional Review Board of the Health, Rehabilitation, Integration, and Research Center (HRIR), and all participants were asked to submit an online informed consent detailing the aim of the study and highlighting that their participation was voluntary before entering the study survey interface. All data were collected anonymously and handled confidentially, adhering to the principles outlined in the Declaration of Helsinki [22].

### Data collection

Participants were recruited from ten rehabilitation centers across the eight Lebanese governorates. A researcher visited the centers to explain the study’s objectives and procedures to eligible individuals. Those who expressed interest were invited to participate and provided informed consent by electronically signing a consent form before proceeding.

The study questionnaire was administered via a Google Form, distributed to participants through their preferred social media platforms. Participants who required assistance in completing the survey due to their physical or cognitive limitations were helped by the researcher or a trusted family member/caregiver. The questionnaire was structured into three sections:

1. Sociodemographic Characteristics: This section collected data on participants’ age, gender, marital status, and employment status before and after their disability.
2. Clinical Characteristics: This included questions about the type and cause of the disability, and its level was assessed using the Arabic version of the Katz Index of Independence in Activities of Daily Living (ADL).
3. Outcome Measures: This section contained the Arabic Community Integration Questionnaire (AR-CIQ) and the Arabic 12-Item Short-Form quality of life measure.

The average time for completing the questionnaire was 15–20 minutes. Out of 165 potential participants who were approached, 150 completed the survey, resulting in a 90.9% response rate.

### The Katz index of independence in activities of daily living (ADL)

The Katz Index is a widely used instrument that evaluates an individual’s independence in performing six basic activities of daily living: bathing, dressing, transferring, toileting, feeding, and continence [23]. The tool is scored dichotomously, with 1 point assigned for independence in an activity and 0 for requiring any assistance. The total score ranges from 0 to 6, where a higher score indicates greater independence [24]. This tool has been translated and validated into various languages, including Brazilian, Arabic, Turkish, and Persian [25–27]. For this study, we utilized a previously translated and validated Arabic version of the tool for the Lebanese population. This version has demonstrated strong split-half reliability: the first three subscales (bathing, dressing, and toileting) had a high internal consistency with a Cronbach’s alpha of 0.90, while the second three subscales (transferring, continence, and feeding) had a Cronbach’s alpha of 0.65. The correlation between the two halves was also strong (r = 0.8), and the tool is a valid measure for assessing daily activities among a Lebanese elderly population. The validity of this Arabic ADL version was further examined using several methods. The tool showed high sensitivity and a high negative predictive value, indicating that it is highly effective at correctly identifying individuals who are not dependent. Conversely, its specificity and positive predictive value were low, meaning it was less effective at correctly identifying dependent individuals. A low, non-significant negative correlation was found between age and ADL score (r = −0.04, p = 0.57), suggesting a trend where increased age is associated with greater functional dependence. Additionally, the tool did not show a significant ability to discriminate between different cognitive impairment groups (p = 0.26) or between different age groups (p = 0.86), though the oldest group descriptively had lower ADL scores [28].

### The Arabic community integration questionnaire (AR-CIQ)

The Community Integration Questionnaire (CIQ) is a 15-item tool that assesses an individual’s involvement in three key areas of community life: home integration, social integration, and productive activities [15]. The CIQ has been evaluated and used in different populations, including adults with a physical disability [14], spinal cord injury [29], and multiple sclerosis [17]. Higher scores on the CIQ indicate better community integration. The CIQ uses a fixed-point scale for scoring across its 15 items, with the total score determined by summing all individual item scores to yield a maximum possible score of 27 points. This total score is divided among the three subscales: Home Integration (max 10 points), Social Integration (max 10 points), and Productive Activities (max 7 points). Higher scores indicate a greater degree of community integration and independence. Conversely, low scores signify poorer overall community integration and restricted participation in life situations; specifically, a low score in Home Integration suggests greater dependence on others for domestic tasks, a low score in Social Integration indicates limited social engagement, and a low score in Productive Activities reflects minimal involvement in roles such as work, school, or volunteering [15]. For this study, we used a translated and culturally adapted Arabic version of the tool (AR-CIQ), which underwent validation as part of the present research. The total score is calculated from the sum of the individual item scores, which in the original CIQ, are typically on a 0 − 2 or 0 − 3 scale for each item.

### The Arabic 12-item short-form health survey (SF-12)

The SF-12, a summarized version of the 36-item Short Form Health Survey (SF-36), was used to examine the health-related quality of life. For this study, we used a previously validated Arabic version of the SF-12 [30], which is a reliable and valid tool for the Lebanese population. Its psychometric properties were confirmed with a satisfactory internal consistency, showing a Cronbach’s alpha of 0.743 for the PCS and 0.707 for the MCS. Principal component analysis supported a two-factor solution (physical and mental), which explained a total variance of 55.75%. This analysis confirmed the tool’s construct validity, with specific subscales (physical functioning, role physical, bodily pain, and general health) loading highly onto the PCS, and others (vitality, social functioning, role emotional, and mental health) associating more with the MCS [31].

### Statistical analysis

All statistical analyses were conducted using **SPSS version 26.0** for Windows, with a significance level set at **p < 0.05**. Descriptive statistics, including frequencies, percentages, means, and standard deviations, were used to summarize the socio-demographic, clinical, and outcome measure characteristics of the study participants.

### Outcome measures scoring

- **Arabic Community Integration Questionnaire (AR-CIQ):** Total and subscale scores for Home Integration, Social Integration, and Productivity were calculated by summing the scores of the corresponding items. A higher score indicates a greater level of community integration.
- **Arabic 12-Item Short-Form Health Survey (SF-12):** This tool’s items were used to generate two main scores: the Physical Component Summary (PCS) and the Mental Component Summary (MCS), using a standard scoring algorithm. Higher scores on both components indicate a better health-related quality of life.
- **Katz Index of Independence in ADL:** A total score was computed by summing the scores of the six items. This continuous variable, ranging from 0 to 6, was used in the analysis, with a higher score indicating greater independence.

### Validity and Reliability

To evaluate construct validity, the underlying factor structure of the AR-CIQ was explored using Exploratory Factor Analysis (EFA) with Principal Component Analysis (PCA) and varimax rotation. In terms of internal consistency, the reliability of each subscale was assessed using Cronbach’s alpha, with a value greater than 0.70 considered satisfactory [32]. The relationships between the AR-CIQ scores and the SF-12 quality of life dimensions and the Katz Index of Independence in ADL were examined using Pearson’s correlation coefficient (r) to examine convergent validity. The strength of these correlations was interpreted based on Cohen’s criteria: weak (r < 0.30), moderate (0.30 ≤ r < 0.50), or strong (r > 0.50) [33].

To identify the factors associated with community integration, we performed a series of four separate Binary Logistic Regression analyses, one for each of the new subscales identified in the Exploratory Factor Analysis: Social and Leisure Integration, External Integration, Domestic Integration, and Social Support Integration. Odds ratio was used to measure the association between each dimension and the different factors.

## Results

### Baseline characteristics

“S1 Table” displays the baseline characteristics of the study participants. The study sample consisted of 150 individuals with physical disabilities, with 86 males (57.3%) and 64 females (42.7%). In terms of educational achievement, 30.7% had secondary education, 21.3% had high school diplomas, 27.3% had university degrees, and 1.3% were postgraduates. Regarding employment, 50% of participants were employed before their injury, with this proportion reducing to 31.3% post-injury. 60% of the participants were unmarried.

The participants presented with a range of physical disabilities, including unilateral lower limb amputation (10.0%), unilateral upper limb amputation (6.0%), bilateral lower limb amputation (5.3%), traumatic brain injury (12.0%), stroke (19.3%), spinal cord injury (14.0%), multiple sclerosis (10.0%), cerebral palsy (15.3%), and spina bifida (8.0%). The duration since the onset of physical disability varied significantly among participants. Notably, 44.0% had experienced their disability for ten years, while 22.7% had been with disability for one to three years.

Regarding independence in activities of daily living, participants had a mean score of 2.27 ± 2.04 out of 6 on the Activities of Daily Living (ADL) Scale, indicating a significant level of need for assistance in daily tasks. Community integration, as measured by the AR-CIQ, showed a mean score of 7.91 ± 2.17 for home integration, suggesting moderate integration into the home environment; 7.59 ± 1.64 for social integration, reflecting reasonable social engagement; and 2.82 ± 2.22 for integration into productive activities, indicating limited participation in productive roles. The total CIQ score was 18.32 ± 3.17. The SF-12 scores revealed a PCS-12 score of 37.16 ± 5.60, indicating physical health challenges, and an MCS-12 score of 40.19 ± 10.44, suggesting a prevalence of clinical depression among participants.

### Construct validity

An Exploratory Factor Analysis (EFA) was conducted on the sample to investigate the factor structure of the AR-CIQ using PCA with varimax rotation. The Kaiser-Meyer-Olkin (KMO) measure and Bartlett’s test of sphericity assessed sampling adequacy. The number of factors to retain was determined based on eigenvalues greater than 1, with items being eliminated if they had low communalities (<0.4) or high cross-loadings [34].

A four-factor solution was extracted with eigenvalues of 4.409, 2.151, 1.261, and 1.053, which collectively accounted for 68.27% of the total variance. The communalities ranged from 0.50 for item 9 of Scale 1 (CIQ 9) to 0.85 for item 2 (see S2 Table). The KMO measure was 0.80, exceeding the commonly accepted threshold of 0.60, indicating strong sampling adequacy [35]. Bartlett’s test of sphericity was highly significant (χ2 = 658.782, df = 78, p < 0.0001), suggesting that the variables are significantly correlated and suitable for factor analysis.

The final analysis included 13 items due to the aggregation of items 13–15 into a single category (job/school). Although the factor structure did not replicate the original scale similarly, it maintained notable consistencies, especially concerning home and social integration.

**Fig 1:**
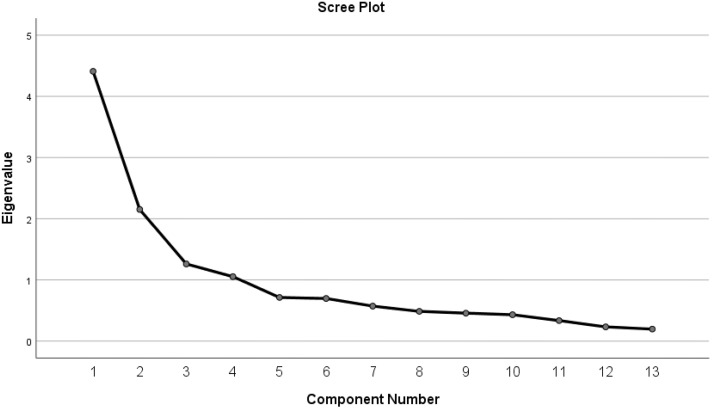
Scree Plot representing the four-factor structure.

Factor 1, which included items 1, 7, 8, and 9, was labeled “social outdoor integration,” incorporating elements from both the home integration and social integration subscales of the original scale. Factor 2, consisting of items 5, 6, 12, and 13, was identified as “ productive and social management,” combining aspects of external arrangements from social integration with social and financial arrangements from productive activities. Factor 3, represented by items 2, 3, and 4, focused on “domestic integration,” emphasizing home-related activities. Finally, Factor 4, including items 10 and 11, reflected “social support” elements. A detailed description of the EFA results is shown in “S2 Table”. The inspection of the scree plot provided visual evidence for the multidimensionality of the scale, confirming the four-factor solution as displayed in Fig 1.

### Reliability analysis

The reliability of the AR-CIQ was assessed using Cronbach’s alpha coefficients of its resulting subscales. The domestic integration subscale demonstrated excellent internal consistency (α = 0.830). The productive and social management and social support integration subscales showed moderate reliability (α = 0.611 and 0.621, respectively). The social outdoor integration subscale presented low reliability (α = 0.333). However, removing item 1 significantly improved reliability to α = 0.721 “S3 Table”.

### Correlation of CIQ subscales and QOL dimensions

The correlation analyses identified significant relationships between the physical and mental quality of life dimensions and the Arabic CIQ scores. The correlation between the Community Integration total score and PCS12 was found to be positive and significant, with a correlation coefficient of 0.197 (p < 0.05), indicating a weak but statistically significant relationship. Regarding CIQ total score and MCS12, a statistically significant correlation was demonstrated with a correlation coefficient of 0.418 (p < 0.01). This finding implies a moderate positive relationship between community integration and the mental health dimension of the SF-12.

Social outdoor integration was moderately positively correlated with MCS-12 scores (r = 0.261, p = 0.001). The productive and social management subscale showed a significant positive correlation with both PCS-12 (r = 0.293, p = 0.001) and MCS-12 (r = 0.424, p < 0.001), showing its association with quality of life. Similarly, the social support subscale had significant positive correlations with PCS-12 (r = 0.226, p = 0.005) and MCS-12 (r = 0.471, p < 0.001), indicating a moderate relationship with quality of life. Conversely, domestic integration did not exhibit significant correlations with PCS-12 (r = 0.101, p = 0.217) or MCS-12 (r = −0.099, p = 0.229), suggesting no significant relationship to quality of life dimensions “S4 Table”.

### Factors associated with CIQ subscales

“S5 Table” depicts the factors associated with the different community integration subscales. In terms of social outdoor integration, males demonstrate significantly higher odds of achieving better integration compared to females, with an odds ratio (OR) of 1.60 (95% CI: 1.23–2.09, p < 0.0001). For productive and social management integration, employed individuals show higher odds of better integration than their unemployed counterparts, with an OR of 6.43 (95% CI: 3.37–12.25, p < 0.0001). Conversely, those with SCI experience significantly worse productive and social management integration, as indicated by an OR of 0.21 (95% CI: 0.08–0.55, p = 0.002). Regarding domestic integration, females have significantly better integration than males, with an OR of 0.24 (95% CI: 0.17–0.34, p < 0.0001). Additionally, married individuals exhibit higher odds of better domestic integration than unmarried individuals, with an OR of 1.94 (95% CI: 1.24–3.05, p = 0.004). Participants with amputation, SCI, and MS have poorer domestic integration relative to other conditions. Furthermore, individuals with traumatic injuries have better domestic integration compared to those with non-traumatic injuries, with an OR of 1.89 (95% CI: 1.22–2.92, p = 0.004). Lower ADL scores are also significantly associated with poorer domestic integration, with an OR of 0.55 (95% CI: 0.37–0.81, p = 0.003). For social support integration, employed individuals exhibit significantly better integration compared to unemployed individuals, with an OR of 1.76 (95% CI: 1.15–2.68, p = 0.008).

## Discussion

The present study aimed to translate and validate the AR-CIQ among a sample of Lebanese adults with physical disabilities while also exploring factors associated with its dimensions. The findings demonstrate that the AR-CIQ exhibits reasonable psychometric properties within this population, supporting its utility as a culturally adapted tool for assessing community integration. However, the factor structure of the AR-CIQ diverged from the original CIQ, reflecting unique cultural and contextual influences on the conceptualization of community integration in the Lebanese context.

### Reliability

The reliability of the AR-CIQ subscales was assessed using Cronbach’s alpha coefficients. Our findings for the Home Integration subscale demonstrated excellent reliability with a Cronbach’s alpha of 0.830, which is consistent with similar international studies, such as the Croatian CIQ-R, which reported an alpha of 0.72 [36], and the original CIQ, which found an alpha of 0.84 in a sample of individuals with physical disabilities [14]. The productive and social Management Subscale and Social Support subscale also showed acceptable reliability (α = 0.611 and α = 0.621, respectively), aligning with the results of earlier research [14,29,36]. While the Social Integration subscale had the lowest reliability in our study (alpha = 0.333), this finding is not unique. The subscale has been consistently problematic across various international validations [11,19], suggesting that its items may not fully capture the complex, multi-dimensional nature of social integration across different cultures. This low internal consistency is a crucial psychometric indicator of the cultural and contextual divergence noted in our factor analysis. In Lebanon, the close-knit, family- and community-centric nature of society blurs the boundaries that define distinct social, domestic, and productive roles in a way that differs from the Western context of the original CIQ. Despite this known limitation, we retained the subscale due to its significant conceptual importance in the field of rehabilitation.

### Factor structure and construct validity

Exploratory factor analysis revealed a four-factor structure for the AR-CIQ, which differed from the original three-factor model of the CIQ. This divergence aligns with previous studies that have shown the construct of community integration is significantly influenced by cultural and contextual factors [37–39]. The AR-CIQ’s factor solution reflects the interconnectedness of social, productive, and domestic roles in Lebanese culture. The finding that the factor structure of the AR-CIQ did not precisely replicate the three-factor model of the original CIQ is a significant result of this study. This discrepancy, which is common in cross-cultural validation research, can be attributed to the unique local and cultural context in which the AR-CIQ was administered. While the original CIQ was developed in a Western context, where concepts of social and productive integration may be distinct [40]. These domains are often perceived differently in the Lebanese cultural setting. The close-knit, family- and community-centric nature of Lebanese society may blur the boundaries between what is considered “social” versus “productive” or “home” integration [37]. Our exploratory factor analysis, which yielded a four-factor solution, provides a scientific basis for this observation.

For instance, our analysis identified a factor called “Social Outdoor Integration,” which combined items related to social activities with grocery shopping. This highlights how, in the Lebanese cultural context, outdoor activities serve a dual purpose as both practical tasks and opportunities for social engagement. This finding is consistent with prior research on individuals with physical disabilities, where the need for assistance can blur the lines between home and social integration [14].

Factor 2, **“**Productive and Social Management**,”** integrated items related to work, education, and social planning. This reflects the cultural emphasis on how productivity is not an isolated pursuit but a means of fulfilling social expectations and contributing to the community. Items related to travel and school activities were included in this factor, aligning with findings that travel and work are often seen as social actions, regardless of their primary purpose [14]. In this context, managing one’s education and work is viewed as a form of broader community integration, where fulfilling productive roles is integral to participating fully in social life.

The factor for Domestic Integration closely mirrored the original home integration subscale, emphasizing the centrality of household responsibilities in a collectivist society like Lebanon. Finally, a new factor, **“**Social Support**,”** emerged, highlighting the crucial role of support networks in facilitating community integration, a finding consistent with research on collectivist cultures [41].

In summary, the four-factor structure of the AR-CIQ reflects the unique cultural dimensions of community integration in Arabic-speaking populations. While this factor solution differs from the original CIQ, it maintains the core elements of home integration and social support. This indicates that while the fundamental constructs of community integration remain relevant across cultural contexts, their expression and organization within a measurement tool must be adapted to reflect the local context [14,37,41]. This divergence in factor structure suggests that a simple translation is not sufficient; a cultural adaptation is necessary to ensure the instrument accurately measures the intended constructs. Our findings reinforce the importance of rigorous psychometric validation in new cultural settings.

### Convergent validity and associations with quality of life

The AR-CIQ demonstrated convergent validity through its significant, but weak to moderate, correlations with different SF-12 subscales. These findings suggest that while community integration and quality of life are related, they remain distinct constructs. For instance, some correlations were very weak (e.g., CIQ with PCS12: r = 0.197), and it is important to note that while statistically significant, a correlation of this limited strength has a very small effect size and may not have a meaningful clinical or practical impact.

Furthermore, we found a lack of a significant relationship between home integration and quality of life (both physical and psychological), a finding that aligns with a previous study [14]. This may be since the home environment for individuals with physical disabilities often represents a stable and controlled space where daily routines are well-established. As such, variations in the home environment might not be strongly correlated with broader, more comprehensive measures of quality of life, which are likely more influenced by external factors like social participation and access to resources.

The strongest correlation was observed between the external integration subscale and the mental component of the SF-12, indicating that enhancing community integration could be a viable strategy for improving mental quality of life in this population [42]. These results align with previous studies [14,29,43], reinforcing the relevance of the AR-CIQ as a tool for assessing community integration in Arabic-speaking populations.

### Demographic and disability-related factors

The community integration scores revealed moderate integration into the home environment, reasonable social engagement, and limited participation in productive activities. These findings align with previous research, indicating that individuals with physical disabilities often face challenges in achieving full community integration, particularly in the domains of social and productive activities [44].

The study identified several factors associated with community integration, including gender, employment status, and type of disability. Males exhibited better social and leisure integration compared to females, while employed individuals reported higher levels of external integration than their unemployed counterparts [45]. These findings highlight the multifaceted nature of community integration and the need for targeted interventions that address gender- and employment-related disparities. Additionally, individuals with spinal cord injuries demonstrated poorer integration outcomes, underscoring the necessity for tailored support mechanisms to enhance their participation in community life [46].

The participants’ educational attainment varied, with a substantial portion having completed secondary education or higher, which aligns with findings suggesting that higher education levels are often associated with better health outcomes and quality of life [47]. The employment status of participants before and after their injuries reveals a concerning trend. While 50% were employed before their disability, this figure declined to 31.3% post-injury. This reduction aligns with existing literature, which highlights the significant barriers individuals with disabilities face in securing and maintaining employment. These barriers not only contribute to financial instability but also exacerbate social isolation and diminish overall quality of life

The decline in employment among participants, from 50% pre-injury to 31.3% post-injury, reflects the significant barriers individuals with disabilities face in the labor market. Specifically in Lebanon, studies have shown that individuals with disabilities are significantly less likely to participate in the labor force, reinforcing the need for policies that enhance both employment opportunities and social [48]. This trend aligns with existing literature, which highlights how unemployment can influence social isolation and reduce the overall quality of life in people with disabilities [49,50].

Quality of life assessments further underscore the challenges faced by participants, with PCS-12 and MCS-12 scores indicating physical health challenges and a prevalence of clinical depression, respectively. These findings are consistent with existing literature that highlights the mental health struggles often encountered by individuals with disabilities, particularly those with long-term conditions [51].

In conclusion, this study highlights the intricate relationship between physical disabilities, community integration, and quality of life, emphasizing the pressing need for tailored interventions that enhance both social participation and mental well-being. The findings reinforce the importance of culturally adapted assessment tools, such as the AR-CIQ, in accurately capturing the unique experiences of individuals with disabilities in Lebanon.

### Limitations

This study has several limitations that should be acknowledged. First, the sample size of 150 individuals may limit the generalizability of the findings to the broader population of individuals with physical disabilities in Lebanon and other Arabic-speaking regions. Additionally, the cross-sectional design prevents the establishment of causal relationships between community integration, quality of life, and associated factors, highlighting the need for longitudinal studies. The reliance on self-reported measures introduces potential recall and social desirability biases, which could be mitigated by incorporating objective assessments.

Furthermore, while the AR-CIQ demonstrated strong psychometric properties, its factor structure deviated from the original CIQ, reflecting cultural influences on the conceptualization of community integration and potentially limiting comparability with international studies. A key limitation is the use of PCA to establish the factor structure of the AR-CIQ. While PCA is useful for data reduction, it is not a pure common factor model and may not be the optimal choice for identifying underlying latent constructs. We selected this method because our primary aim was an exploratory cross-cultural validation without a pre-existing hypothesis regarding the factor structure. A larger sample size would be required to perform a robust confirmatory factor analysis (CFA) to validate our findings.

The analysis also did not explicitly address the extent of multicollinearity or the association between the factors themselves. The results may have also been influenced by sample characteristics, such as unbalanced distribution in terms of disability type and duration of injury. We also did not account for environmental and policy-related barriers, which are critical determinants of community integration. Moreover, while mental health challenges were identified, pre-existing conditions were not controlled for, which may have influenced the reported associations. Lastly, given the diversity of disability types within the sample, further research is needed to explore how different conditions uniquely impact integration and quality of life. Despite these limitations, the study provides valuable insights into the adaptation and validation of the AR-CIQ and underscores the importance of contextually relevant measures for assessing community integration in Lebanon.

### Implications for practice and research

This study has several strengths that contribute to its significance in the field of rehabilitation and disability research. First, it is among the few studies to validate a culturally adapted measure of community integration in an Arabic-speaking population, addressing a critical gap in the literature. By translating and validating the AR-CIQ, the study ensures that community integration is assessed in a manner that aligns with the social and cultural context of Lebanon, enhancing its applicability in both clinical and research settings. Additionally, the study provides a comprehensive analysis of the factors influencing community integration, offering valuable insights into the interplay between disability, social participation, and quality of life. The findings have important implications for both practice and policy, emphasizing the need for targeted interventions that promote employment opportunities, enhance social support, and improve mental health outcomes for individuals with physical disabilities. Moreover, the identification of a distinct factor structure for the AR-CIQ highlights the influence of cultural and environmental factors on community integration, underscoring the necessity for context-specific approaches in disability research.

These insights can inform future research exploring how different cultural contexts shape integration experiences and how rehabilitation strategies can be tailored to better support individuals with disabilities. Lastly, by identifying the disparities in integration outcomes based on gender, employment status, and disability type, the study provides a foundation for developing inclusive policies and rehabilitation programs that address these challenges and foster greater community participation.

## Conclusion

This study successfully translated and validated the Arabic version of the AR-CIQ among Lebanese adults with physical disabilities, demonstrating its psychometric robustness and cultural relevance. The findings support the AR-CIQ as a reliable and valid tool for assessing community integration in Arabic-speaking populations, though its factor structure diverged from the original CIQ, reflecting cultural and contextual influences. The identified four-factor structure underscores the interconnected nature of home, social, and productive integration within the Lebanese context. Moreover, the significant associations between community integration and quality of life highlight the need for targeted interventions that enhance participation and well-being. By providing a culturally adapted measure, this study contributes to advancing disability research and rehabilitation practices in the region. Future studies should further explore the applicability of the AR-CIQ across different Arabic-speaking populations and evaluate its responsiveness to rehabilitation interventions over time.

## Supporting information

S1 Table Participant’s Characteristics. (DOCX)

S2 Table Exploratory factor analysis of the AR-CIQ. (DOCX)

S3 Table Reliability analysis. (DOCX)

S4 Table Correlation between CIQ and QOL subscales. (DOCX)

S5 Table Factors associated with community integration subscales. (DOCX)

## References

[1] Gretschel D, Visagie S, Inglis G. Community integration of adults with disabilities post discharge from an in-patient rehabilitation unit in the Western Cape. S Afr J Physiother. 2017;73(1):361. doi: 10.4102/sajp.v73i1.361 30135906 PMC6093139

[2] Vargus-Adams JN, Majnemer A. International Classification of Functioning, Disability and Health (ICF) as a framework for change: revolutionizing rehabilitation. J Child Neurol. 2014;29(8):1030–5. doi: 10.1177/0883073814533595 24850572

[3] Organization WH. International Classification of Functioning, Disability and Health: ICF. Geneva: WHO. 2001.

[4] Kashif M, Jones S, Darain H, Iram H, Raqib A, Butt AA. Factors influencing the community integration of patients following traumatic spinal cord injury: a systematic review. J Pak Med Assoc. 2019;69(9):1337–43. 31511721

[5] Heeb R, Putnam M, Keglovits M, Weber C, Campbell M, Stark S, et al. Factors influencing participation among adults aging with long-term physical disability. Disabil Health J. 2022;15(1):101169. doi: 10.1016/j.dhjo.2021.101169 34332950 PMC10686630

[6] Sander AM, Clark A, Pappadis MR. What is community integration anyway?: defining meaning following traumatic brain injury. J Head Trauma Rehabil. 2010;25(2):121–7. doi: 10.1097/HTR.0b013e3181cd1635 20134333

[7] McColl MA, Carlson P, Johnston J, Minnes P, Shue K, Davies D, et al. The definition of community integration: perspectives of people with brain injuries. Brain Inj. 1998;12(1):15–30. doi: 10.1080/026990598122827 9483334

[8] Wood-Dauphinee SL, Opzoomer MA, Williams JI, Marchand B, Spitzer WO. Assessment of global function: The Reintegration to Normal Living Index. Arch Phys Med Rehabil. 1988;69(8):583–90. 3408328

[9] McColl MA, Davies D, Carlson P, Johnston J, Minnes P. The community integration measure: development and preliminary validation. Arch Phys Med Rehabil. 2001;82(4):429–34. doi: 10.1053/apmr.2001.22195 11295000

[10] Walker N, Mellick D, Brooks CA, Whiteneck GG. Measuring participation across impairment groups using the Craig Handicap Assessment Reporting Technique. Am J Phys Med Rehabil. 2003;82(12):936–41. doi: 10.1097/01.PHM.0000098041.42394.9A 14627930

[11] Willer B, Rosenthal M, Kreutzer JS, Gordon WA, Rempel R. Assessment of community integration following rehabilitation for traumatic brain injury. J Head Trauma Rehabilitation. 1993;8(2):75–87. doi: 10.1097/00001199-199308020-00009

[12] Turcotte S, Beaudoin M, Vallée C, Vincent C, Routhier F. Psychometric properties of the Community Integration Questionnaire: a systematic review of five populations. Clin Rehabil. 2019;33(11):1775–87. doi: 10.1177/0269215519867998 31397182

[13] Ahmed N, Quadir MM, Rahman MA, Alamgir H. Community integration and life satisfaction among individuals with spinal cord injury living in the community after receiving institutional care in Bangladesh. Disabil Rehabil. 2018;40(9):1033–40. doi: 10.1080/09638288.2017.1283713 28637130

[14] Hirsh AT, Braden AL, Craggs JG, Jensen MP. Psychometric properties of the community integration questionnaire in a heterogeneous sample of adults with physical disability. Arch Phys Med Rehabil. 2011;92(10):1602–10. doi: 10.1016/j.apmr.2011.05.004 21851927 PMC3371822

[15] Sander AM, Fuchs KL, High WM Jr, Hall KM, Kreutzer JS, Rosenthal M. The Community Integration Questionnaire revisited: an assessment of factor structure and validity. Arch Phys Med Rehabil. 1999;80(10):1303–8. doi: 10.1016/s0003-9993(99)90034-5 10527092

[16] Ioncoli M, Berardi A, Tofani M, Panuccio F, Servadio A, Valente D, et al. Crosscultural Validation of the Community Integration Questionnaire-Revised in an Italian Population. Occup Ther Int. 2020;2020:8916541. doi: 10.1155/2020/8916541 32934614 PMC7481919

[17] Negahban H, Fattahizadeh P, Ghasemzadeh R, Salehi R, Majdinasab N, Mazaheri M. The Persian version of Community Integration Questionnaire in persons with multiple sclerosis: translation, reliability, validity, and factor analysis. Disabil Rehabil. 2013;35(17):1453–9. doi: 10.3109/09638288.2012.741653 23330593

[18] Xie HX, Zhang Q, Wei Y, Li N, Wu AR, Zeng XH. Validation study of the Chinese version of the Community Integration Questionnaire-Revised for individuals with spinal cord injury in Mainland China. J Spinal Cord Med. 2023;:1–9. 10.1080/10790268.2023.2217589 PMC11533259 37428443

[19] Rintala DH, Novy DM, Garza HM, Young ME, High WM, Chiou-Tan FY. Psychometric properties of a Spanish-language version of the Community Integration Questionnaire (CIQ). Rehabilitation Psychol. 2002;47(2):144–64. doi: 10.1037/0090-5550.47.2.144

[20] Beaton DE, Bombardier C, Guillemin F, Ferraz MB. Guidelines for the process of cross-cultural adaptation of self-report measures. Spine (Phila Pa 1976). 2000;25(24):3186–91. 11124735 10.1097/00007632-200012150-00014

[21] Floyd FJ, Widaman KF. Factor analysis in the development and refinement of clinical assessment instruments. Psychological Assessment. 1995;7(3):286–99. doi: 10.1037/1040-3590.7.3.286

[22] Williams JR. The Declaration of Helsinki and public health. Bull World Health Organ. 2008;86(8):650–2. doi: 10.2471/blt.08.050955 18797627 PMC2649471

[23] Katz S, Ford AB, Moskowitz RW, Jackson BA, Jaffe MW. Studies of illness in the aged. The index of ADL: a standardized measure of biological and psychosocial function. JAMA. 1963;185:914–9. 14044222 10.1001/jama.1963.03060120024016

[24] Katz S, Downs TD, Cash HR, Grotz RC. Progress in development of the index of ADL. Gerontologist. 1970;10(1):20–30. doi: 10.1093/geront/10.1_part_1.20 5420677

[25] Arik G, Varan HD, Yavuz BB, Karabulut E, Kara O, Kilic MK, et al. Validation of Katz index of independence in activities of daily living in Turkish older adults. Arch Gerontol Geriatr. 2015;61(3):344–50. doi: 10.1016/j.archger.2015.08.019 26328478

[26] Pashmdarfard M, Azad A. Assessment tools to evaluate Activities of Daily Living (ADL) and Instrumental Activities of Daily Living (IADL) in older adults: A systematic review. Med J Islam Repub Iran. 2020;34:33. doi: 10.34171/mjiri.34.33 32617272 PMC7320974

[27] Azad A, Mohammadinezhad T, Taghizadeh G, Lajevardi L. Clinical assessment of activities of daily living in acute stroke: Validation of the Persian version of Katz Index. Med J Islam Repub Iran. 2017;31:30. doi: 10.18869/mjiri.31.30 29445659 PMC5804429

[28] Nasser R, Doumit J. Validity and reliability of the Arabic version of activities of daily living (ADL). BMC Geriatr. 2009;9:11. doi: 10.1186/1471-2318-9-11 19327172 PMC2670307

[29] Kratz AL, Chadd E, Jensen MP, Kehn M, Kroll T. An examination of the psychometric properties of the community integration questionnaire (CIQ) in spinal cord injury. J Spinal Cord Med. 2015;38(4):446–55. doi: 10.1179/2045772313Y.0000000182 24621050 PMC4612200

[30] Müller-Nordhorn J, Roll S, Willich SN. Comparison of the short form (SF)-12 health status instrument with the SF-36 in patients with coronary heart disease. Heart. 2004;90(5):523–7. doi: 10.1136/hrt.2003.013995 15084550 PMC1768233

[31] Haddad C, Sacre H, Obeid S, Salameh P, Hallit S. Validation of the Arabic version of the “12-item short-form health survey” (SF-12) in a sample of Lebanese adults. Arch Public Health. 2021;79(1):56. doi: 10.1186/s13690-021-00579-3 33892801 PMC8067286

[32] Tavakol M, Dennick R. Making sense of Cronbach’s alpha. Int J Med Educ. 2011;2:53–5. doi: 10.5116/ijme.4dfb.8dfd 28029643 PMC4205511

[33] Schober P, Boer C, Schwarte LA. Correlation Coefficients: Appropriate Use and Interpretation. Anesth Analg. 2018;126(5):1763–8. doi: 10.1213/ANE.0000000000002864 29481436

[34] Maskey R, Fei J, Nguyen H-O. Use of exploratory factor analysis in maritime research. Asian J Shipping and Logistics. 2018;34(2):91–111. doi: 10.1016/j.ajsl.2018.06.006

[35] Beavers AS, Lounsbury JW, Richards JK, Huck SW, Skolits G, Esquivel SL. Practical considerations for using exploratory factor analysis in educational research. Practical Assessment Res Evaluation. 2013;18:1–13.

[36] Willer B, Ottenbacher KJ, Coad ML. The community integration questionnaire. A comparative examination. Am J Phys Med Rehabil. 1994;73(2):103–11. doi: 10.1097/00002060-199404000-00006 8148099

[37] Salter K, Foley N, Jutai J, Bayley M, Teasell R. Assessment of community integration following traumatic brain injury. Brain Inj. 2008;22(11):820–35. doi: 10.1080/02699050802425428 18850341

[38] Lequerica AH, Chiaravalloti ND, Sander AM, Pappadis MR, Arango-Lasprilla JC, Hart T, et al. The Community Integration Questionnaire: factor structure across racial/ethnic groups in persons with traumatic brain injury. J Head Trauma Rehabil. 2013;28(6):E14-22. doi: 10.1097/HTR.0b013e31826e3ca8 23249771

[39] Sander AM, Clark A, Pappadis MR. What is community integration anyway?: defining meaning following traumatic brain injury. J Head Trauma Rehabil. 2010;25(2):121–7. doi: 10.1097/HTR.0b013e3181cd1635 20134333

[40] Fraga-Maia HMS, Werneck G, Dourado I, Fernandes R de CP, Brito LL. Translation, adaptation and validation of “Community Integration Questionnaire”. Cien Saude Colet. 2015;20(5):1341–52. doi: 10.1590/1413-81232015205.08312014 26017937

[41] Terry R, Townley G. Exploring the Role of Social Support in Promoting Community Integration: An Integrated Literature Review. Am J Community Psychol. 2019;64(3–4):509–27. doi: 10.1002/ajcp.12336 31116874

[42] Umunnah J, Adegoke B, Uchenwoke C, Igwesi-Chidobe C, Alom G. Impact of community-based rehabilitation on quality of life and self-esteem of persons with physical disabilities and their family members. Global Health Journal. 2023;7(2):87–93. doi: 10.1016/j.glohj.2023.04.001

[43] McVeigh SA, Hitzig SL, Craven BC. Influence of sport participation on community integration and quality of life: a comparison between sport participants and non-sport participants with spinal cord injury. J Spinal Cord Med. 2009;32(2):115–24. doi: 10.1080/10790268.2009.11760762 19569458 PMC2678282

[44] Giummarra MJ, Randjelovic I, O’Brien L. Interventions for social and community participation for adults with intellectual disability, psychosocial disability or on the autism spectrum: An umbrella systematic review. Front Rehabil Sci. 2022;3:935473. doi: 10.3389/fresc.2022.935473 36189003 PMC9397886

[45] Pasin T, Dogruoz Karatekin B. Determinants of social participation in people with disability. PLoS One. 2024;19(5):e0303911. doi: 10.1371/journal.pone.0303911 38768173 PMC11104585

[46] García-Rudolph A, Cusso H, Carbonell C, Lopez S, Pla L, Sabaté M, et al. Community integration after spinal cord injury rehabilitation: Predictors and causal mediators. J Spinal Cord Medicine. :1–12. 10.1080/10790268.2024.2386738 PMC12499539 39133061

[47] Meliza SP, Elfindri E, Ariyanto E, Anas Y. Educational Attainment of People with Disabilities: A Comparison Between Public School and Private Schools. Int J Public Administration Manag Economic Development. 2023;8.

[48] Boutros P, Fakih A. The disability employment paradox in developing countries: recent evidence from Lebanon. Development Studies Res. 2023;10(1). doi: 10.1080/21665095.2023.2244173

[49] Jani R, Alias AA, Tumin M. Persons with disabilities’ education and quality of life: evidence from Malaysia. Int J Inclusive Education. 2020;26(8):753–65. doi: 10.1080/13603116.2020.1726511

[50] Ghasemi M, Sadeghi N, Serpoosh MA. A Study of the Factors Related to the Quality of Life of Students with Disabilities at Kerman Universities. Pajouhan Sci J. 2023;21(3):163–74. doi: 10.61186/psj.21.3.163

[51] Ladwig S, Volz M, Haupt J, Pedersen A, Werheid K. Disentangling the relationships of health-related quality of life, depressive symptoms, disability and social support after stroke: A network analysis. J Affective Disorders Reports. 2025;19:100855. doi: 10.1016/j.jadr.2024.100855

