# Supplementary material for: Validity and reliability of the Arabic community integration questionnaire in a Lebanese sample of adults with physical disability
Source: PLoS One. 2025 Nov 18;20(11):e0336717. doi: 10.1371/journal.pone.0336717 (PMC12626315; doi:10.1371/journal.pone.0336717)
Supplement: S1 Table — (DOCX) [file pone.0336717.s001.docx]

S1 Table. Participant’s characteristics

| **Variable** | **Frequencies** | **Percentage (%)** |
| --- | --- | --- |
| **Gender**  Male  Female | 86  64 | 57.3  42.7 |
| **Educational Level**  Elementary  Secondary  High School  University  Postgraduates | 29  46  32  41  2 | 19.5  30.7  21.3  27.3  1.3 |
| **Employment status before injury**  Employed  Unemployed | 75  75 | 50  50 |
| **Employment status after injury**  Employed  Unemployed | 47  103 | 31.3  68.7 |
| **Marital Status**  Married  Unmarried | 60  90 | 40  60 |
| **Monthly Income**  100-300$  300-500$  500-1000$  1000-2000$  More than 2000$ | 35  41  58  11  5 | 23.3  27.3  38.7  7.3  3.3 |
| **Physical Disability**  Unilateral Lower Limb Amputation  Unilateral Upper Limb Amputation  Bilateral Lower Limb Amputation  Traumatic Brain Injury  Stroke  Spinal Cord Injury  Multiple Sclerosis  Cerebral Palsy  Spina Bifida | 15  9  8  18  29  21  15  23  12 | 10.0  6.0  5.3  12.0  19.3  14.0  10.0  15.3  8.0 |
| **Time since Physical Disability**  Less than one year  1 year - 3 years  3 - 5 years  5 -10 years  More than 10 years | 17  34  18  15  66 | 11.3  22.7  12.0  10.0  44.0 |
|  | **Mean ± SD** | |
| **Age** | 39.09 ± 17.66 | |
| **Activities of Daily Living Scale (0-6)** | 2.27 ± 2.04 | |
| **CIQ Home Integration Subscale Score (0-10)** | 7.91±2.17 | |
| **CIQ Social Integration Subscale Score (0-10)** | 7.59±1.635 | |
| **CIQ Integration into Productive Activities** **Subscale Score (0-7)** | 2.82±2.22 | |
| **CIQ Total Score** | 18.32±3.17 | |
| **Participation Scale Score** | 23.13±12.53 | |
| **PCS-12 (0-100)** | 37.16±5.60 | |
| **MCS-12 (0-100)** | 40.19±10.44 | |
| SD: standard deviation | | |
