# Supplementary material for: Validity and reliability of the Arabic community integration questionnaire in a Lebanese sample of adults with physical disability
Source: PLoS One. 2025 Nov 18;20(11):e0336717. doi: 10.1371/journal.pone.0336717 (PMC12626315; doi:10.1371/journal.pone.0336717)
Supplement: S2 Table — (DOCX) [file pone.0336717.s002.docx]

S2 Table. Exploratory factor analysis of the AR-CIQ

| Kaiser-Meyer-Olkin Measure of Sampling Adequacy. | | 0.800 | | | | |
| --- | --- | --- | --- | --- | --- | --- |
| Bartlett's Test of Sphericity | | Approx. Chi-Square | | 658.782 | | |
|  |  | df | | 78 | | |
|  |  | Sig. | | <0.0001 | | |
|  |  | Total Variance Explained | | 68.27% | | |
| Item |  | Factor 1 | Factor 2 | Factor 3 | Factor 4 | Communalities |
|  | Eigenvalue | 4.409 | 2.151 | 1.261 | 1.053 |  |
|  | Variance Explained | 33.92% | 16.55% | 9.70% | 8.10% |  |
| 1 | Who usually does shopping for groceries or other necessities in your household? | 0.496 |  |  |  | 0.638 |
| 2 | Who usually prepares meals in your household? |  |  | 0.893 |  | 0.848 |
| 3 | In your home who usually does normal everyday housework? |  |  | 0.827 |  | 0.733 |
| 4 | Who usually cares for the children in your home? |  |  | 0.795 |  | 0.696 |
| 5 | Who usually plans social arrangements such as get-togethers with family and friends? |  | 0.512 |  |  | 0.624 |
| 6 | Who usually looks after your personal finances such as banking or paying bills? |  | 0.806 |  |  | 0.691 |
| 7 | Shopping | 0.818 |  |  |  | 0.697 |
| 8 | Leisure activities such as movies, sports, restaurants | 0.862 |  |  |  | 0.794 |
| 9 | Visiting friends or relatives | 0.613 |  |  |  | 0.500 |
| 10 | When you participate in leisure activities, do you usually do this alone or with others? |  |  |  | 0.844 | 0.757 |
| 11 | Do you have a best friend with whom you confide? |  |  |  | 0.698 | 0.595 |
| 12 | How often do you travel outside the home? |  | 0.749 |  |  | 0.687 |
| 13 | JOBSCHOOL |  | 0.736 |  |  | 0.619 |
