# Supplementary material for: Validity and reliability of the Arabic community integration questionnaire in a Lebanese sample of adults with physical disability
Source: PLoS One. 2025 Nov 18;20(11):e0336717. doi: 10.1371/journal.pone.0336717 (PMC12626315; doi:10.1371/journal.pone.0336717)
Supplement: S3 Table — (DOCX) [file pone.0336717.s003.docx]

S3 Table. Reliability analysis

| **Arabic CIQ subscales** | **Cronbach’s Alpha** |
| --- | --- |
| **Social Outdoor Integration** | 0.333 (0.721 if item 1 deleted) |
| **Productive and Social Management** | 0.611 |
| **Domestic Integration** | 0.830 |
| **Social Support** | 0.621 |
