# Supplementary material for: Validity and reliability of the Arabic community integration questionnaire in a Lebanese sample of adults with physical disability
Source: PLoS One. 2025 Nov 18;20(11):e0336717. doi: 10.1371/journal.pone.0336717 (PMC12626315; doi:10.1371/journal.pone.0336717)
Supplement: S4 Table — (DOCX) [file pone.0336717.s004.docx]

S4 Table. Correlation between CIQ and QOL subscales

|  | **Community Integration**  **Total Score** | **Social Outdoor Integration** | **Productive and Social Management** | **Domestic Integration** | **Social Support** |
| --- | --- | --- | --- | --- | --- |
| **PCS12** | 0.197^*^ | 0.026 | 0.293^**^ | 0.101 | 0.226^**^ |
|  | 0.016 | 0.751 | 0.001 | 0.217 | 0.005 |
| **MCS12** | 0.418^**^ | 0.261^**^ | 0.424^**^ | 0.099 | 0.471^**^ |
|  | <0.0001 | 0.001 | <0.0001 | 0.229 | <0.0001 |
