# Supplementary material for: Validity and reliability of the Arabic community integration questionnaire in a Lebanese sample of adults with physical disability
Source: PLoS One. 2025 Nov 18;20(11):e0336717. doi: 10.1371/journal.pone.0336717 (PMC12626315; doi:10.1371/journal.pone.0336717)
Supplement: S5 Table — (DOCX) [file pone.0336717.s005.docx]

S5 Table. Factors associated with community integration subscale

| **Factors** | **Social and leisure integration** | | **External Integration** | | **Domestic Integration** | | **Social Support Integration** | |
| --- | --- | --- | --- | --- | --- | --- | --- | --- |
|  | **OR (95% CI)** | **P-value** | **OR** | **P-value** | **OR** | **P-value** | **OR** | **P-value** |
| **Age**  <35 years  >35 years | 1.16 (0.82-1.63)  1 | 0.38 | 1.87 (0.88-3.97)  1 | 0.10 | 0.77 (0.49-1.19) | 0.24 | 1.49 (0.93-2.40)  1 | 0.09 |
| **Gender**  Male  Female | 1.60 (1.23-2.09)  1 | **<0.0001** | 1.41 (0.81-2.47)  1 | 0.22 | **0.24 (0.17-0.34)**  **1** | **<0.0001** | 1.20 (0.83-1.74)  1 | 0.31 |
| **Marital Status**  Married  Unmarried | 1.21 (0.85-1.71)  1 | 0.27 | 1.33 (0.62-2.84)  1 | 0.45 | **1.94 (1.24-3.05)**  **1** | **0.004** | 0.76 (0.46-1.23)  1 | 0.26 |
| **Educational Level**  <college  >college | 0.87 (0.64-1.18)  1 | 0.38 | 0.88 (0.46-1.69) | 0.70 | 0.98 (0.66-1.47)  1 | 0.95 | 0.90 (0.58-1.38)  1 | 0.64 |
| **Employment Status**  Employed  Unemployed | 1.12 (0.83-1.52)  1 | 0.45 | 6.43 (3.37-12.25)  1 | **<0.0001** | 0.74 (0.49-1.09)  1 | 0.13 | **1.76 (1.15-2.68)**  **1** | **0.008** |
| **Physical Condition**  Amputation  Brain Damage (TBI or stroke)  Spinal Cord Injury  MS  Neurodevelopmental Disorder | 0.96 (0.62-1.49)  0.89 (0.58-1.37)  1.34 (0.86-2.11)  0.78 (0.44-1.38)  1 | 0.87  0.61  0.19  0.39 | 0.41 (0.16-1.03)  0.41 (0.17-0.97)  0.21 (0.08-0.55)  0.38 (0.10-1.34)  1 | **0.05**  **0.04**  **0.002**  0.133 | 0.45 (0.26-0.80)  0.77 (0.44-1.35)  0.47 (0.26-0.85)  0.28 (0.13-0.58)  1 | **0.007**  0.36  **0.01**  **0.001** | 1.46 (0.80-2.69)  1.05 (0.58-1.91)  1.37 (0.73-2.57)  1.48 (0.67-3.28)  1 | 0.21  0.85  0.31  0.32 |
| **Cause of Injury**  Traumatic  Non-Traumatic | 1.14 (0.84-1.56) | 0.380 | 0.83 (0.43+1.63) | 0.60 | 1.89 (1.22-2.92)  1 | **0.004** | 0.99(0.62-1.57)  1 | 0.96 |
| **ADL scale**  <3  >3 | 0.79 (0.59-1.08) | 0.147 | 1.23 (0.64-2.35) | 0.52 | 0.55 (0.37-0.81)  1 | **0.003** | 0.97 (0.64-1.49)  1 | 0.91 |
